# The role of gut microbial β-glucuronidases in carcinogenesis and cancer treatment: a scoping review

**DOI:** 10.1007/s00432-024-06028-2

**Published:** 2024-11-13

**Authors:** Lars E. Hillege, Milou A. M. Stevens, Paulien A. J. Kristen, Judith de Vos-Geelen, John Penders, Matthew R. Redinbo, Marjolein L. Smidt

**Affiliations:** 1 GROW - School for Oncology and Reproduction, Maastricht University, Maastricht, the Netherlands; 2 Department of Surgery, FHML, Maastricht University Medical Center+, Maastricht University, P.O. Box 616, 6200 MD Maastricht, the Netherlands; 3 Division of Medical Oncology, Department of Internal Medicine, Maastricht University Medical Center+, Maastricht, the Netherlands; 4 NUTRIM - School of Nutrition and Translational Research in Metabolism, Maastricht University, Maastricht, the Netherlands; 5 Department of Medical Microbiology, Infectious Diseases and Infection Prevention, Maastricht University Medical Center+, Maastricht, the Netherlands; 6 Departments of Chemistry, Biochemistry & Biophysics, and Microbiology & Immunology, University of North Carolina, Chapel Hill, NC USA

**Keywords:** Glucuronidase, Drug therapy, Carcinoma, Neoplasm, Microbiome

## Abstract

**Introduction:**

The human gut microbiota influence critical functions including the metabolism of nutrients, xenobiotics, and drugs. Gut microbial β-glucuronidases (GUS) enzymes facilitate the removal of glucuronic acid from various compounds, potentially affecting anti-cancer drug efficacy and reactivating carcinogens. This review aims to comprehensively analyze and summarize studies on the role of gut microbial GUS in cancer and its interaction with anti-cancer treatments. Its goal is to collate and present insights that are directly relevant to patient care and treatment strategies in oncology.

**Methods:**

This scoping review followed PRISMA-ScR guidelines and focused on primary research exploring the role of GUS within the gut microbiota related to cancer etiology and anti-cancer treatment. Comprehensive literature searches were conducted in PubMed, Embase, and Web of Science.

**Results:**

GUS activity was only investigated in colorectal cancer (CRC), revealing increased fecal GUS activity, variations in the gut microbial composition, and GUS-contributing bacterial taxa in CRC patients versus controls. Irinotecan affects gastrointestinal (GI) health by increasing GUS expression and shifting gut microbial composition, particularly by enhancing the presence of GUS-producing bacteria, correlating with irinotecan-induced GI toxicities. GUS inhibitors (GUSi) can mitigate irinotecan's adverse effects, protecting the intestinal barrier and reducing diarrhea.

**Conclusion:**

To our knowledge, this is the first review to comprehensively analyze and summarize studies on the critical role of gut microbial GUS in cancer and anti-cancer treatment, particularly irinotecan. It underscores the potential of GUSi to reduce side effects and enhance treatment efficacy, highlighting the urgent need for further research to integrate GUS targeting into future anti-cancer treatment strategies.

**Supplementary Information:**

The online version contains supplementary material available at 10.1007/s00432-024-06028-2.

## Introduction

The human gut microbiota harbor trillions of bacteria that carry out numerous crucial functions including influencing the immune system and directly modulating the metabolism of nutrients, xenobiotics, and drugs (Jandhyala et al. 2015; Pope et al. 2017; Quigley 2013). Within the human gastrointestinal (GI) tract, a vast ecosystem resides with microbial species that predominantly belong to five major bacterial phyla: Bacillota, Bacteroidota, Actinomycetota, Pseudomonadota and Verrucomicrobiota. The bacterial composition and density various along the GI tract, with highest bacterial diversity and density in the colon (Rajilić-Stojanović & de Vos 2014; van den Elsen et al. 2017). While the gut microbiota refers to the community of microorganisms themselves, the gut microbiome encompasses the gut microbiota and their ‘theatre of activity’, which comprises structural elements, metabolites, signaling molecules, and the surrounding environmental parameters (Berg et al. 2020). The gut microbiome is connected with human health (Flier & Mekalanos 2009; Greenblum et al. 2012; Ridaura et al. 2013; Turnbaugh et al. 2006) and yet is an uniquely adaptable component of the body (Turnbaugh et al. 2007). There has been a growing interest in the role of gut microbiome in the development of cancer and anti-cancer treatment. Recent studies indicate that the gut microbiome interacts with chemotherapeutic agents using a variety of mechanisms including immunomodulation, enzymatic degradation, altering drug metabolism profile, reduced microbial diversity, shifting microbial ecology, and the induction of the translocation of bacteria and bacterial products (Aarnoutse et al. 2019; Alexander et al. 2017).

**Fig. 1:**
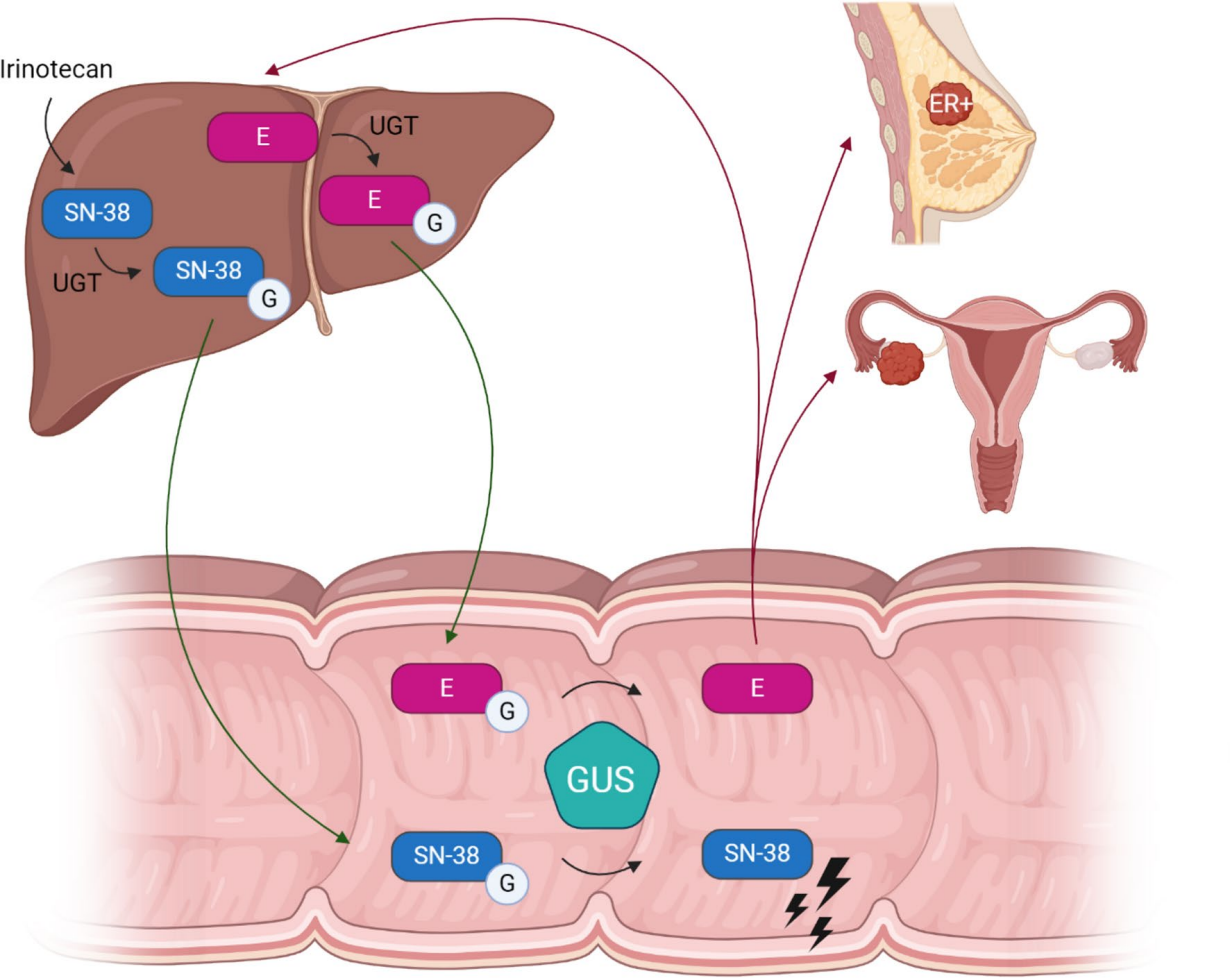
Examples of gut microbial GUS mechanisms associated with cancer. The active metabolite of irinotecan, SN-38, and estrogens are inactivated to SN-38G and estrogen-glucuronides (EG) in the liver by UGT (where the circled G represents glucuronic acid). SN-38G and EG are sent to the intestines via the bile duct. Gut microbial GUS enzymes remove glucuronides from inactivated compounds which leads to reactivation. Reactivated SN-38 generates gut toxicity and irinotecan’s dose-limiting side effect, diarrhea. Reactivated estrogens can be reabsorbed into the bloodstream and may contribute to the development and progression of breast cancer (Nagar & Blanchard 2006; Sui et al. 2021). Created with BioRender.com. E: estrogen; ER: estrogen receptor; G: glucuronic acid; GUS: beta-glucuronidases; UGT: UDP-glucuronosyltransferases.

From these mechanisms, we can deduce that not only the bacteria itself but also enzymes produced by these bacteria can play an important role in interactions between gut microbiota and chemotherapeutic agents. One important group of bacterial enzymes with well described physiological roles is β-glucuronidase (GUS) produced by range of bacteria in the gut microbiota (Pollet et al. 2017). GUS belongs to the glycosidase hydrolase family of enzymes and catalyzes the removal of a glucuronic acid sugar from small molecules and complex carbohydrates (Pollet et al. 2017; Wallace et al. 2015). The diverse gut microbial GUS enzymes have been categorized into eight classes based on structural and functional data (Walker et al. 2022). These analyses have revealed that some classes only act on polysaccharides, others only on small molecules with a single glucuronic acid bound, and some can use both types of substrates (Walker et al. 2022). In terms of the small molecule glucuronides that are substrates for gut microbial GUS proteins, these include drugs (Bhatt et al. 2020; Biernat et al. 2019; Ervin et al. 2019a; Jariwala et al. 2020; Roberts et al. 2013), toxins (Zhang et al. 2022), and a range of endobiotics including hormones (Ervin et al. 2019b; Pellock & Redinbo 2017). Small molecules with glucuronic acid attached are produced by Phase II drug metabolizing UDP glucuronosyltransferase (UGT) enzymes in the liver. These inactive glucuronide conjugates are subsequently sent to the urine and intestines for excretion (Ervin et al. 2019b). While in the GI tract, however, such inactive conjugates can be reactivated by the actions of gut microbial GUS enzymes (Zhang et al. 2022). Thus, the composition and activity of the microbial GUS proteins in the host GI tract play a critical role in the metabolism of various substances in the body, including drugs (Sperker et al. 1997) and hormones (Ervin et al. 2019b; Starek-Świechowicz et al. 2021) (see Fig. 1). Furthermore, GUS catalyzes the hydrolysis of inactive glucuronidated toxins in the GI tract, thus reactivating them and allowing the release of carcinogenic compounds both locally in the intestines as well as systemically after reabsorption (Goldin 1990; Pellock & Redinbo 2017).

The actions of gut microbial GUS have been suggested to increase the risk of cancer development in various parts of the body, such as the colon and breast (Kim & Jin 2001; Kwa et al. 2016; Sui et al. 2021). For example, gut microbial GUS have been hypothesized to facilitate the reabsorption of estrogens into the blood circulation (Baker et al. 2017; Patel et al. 2023). High levels of serum estrogens are related to the development of estrogen receptor-positive (ER +) breast cancer and ovarian cancer, and therefore it can be speculated that GUS has a role in these cancer types (Hu et al. 2023; Kwa et al. 2016). Furthermore, it is known that some gut microbiota-derived GUS variants can reactivate detoxified chemotherapeutic compounds (*e.g.,* SN-38, the active metabolite of irinotecan) in the bowel, which might cause severe mucosal damage and diarrhea in colorectal and pancreas cancer patients (Cheng et al. 2019; Nagar & Blanchard 2006; Wallace et al. 2010). Since GUS plays a crucial role in the gut reactivation of chemotherapy agents like SN-38 and regorafenib (Ervin et al. 2019a), the local inhibition of gut microbial GUS activity has been shown to reduce the intestinal toxicity of these drugs and to improve treatment efficacy in mouse models (Awolade et al. 2020; Bhatt et al. 2020; Takada et al. 1982). Therefore, the use of GUS inhibitors (GUSi) in combination with chemotherapy may improve treatment outcomes by reducing the side effects that are dose-limiting.

This review aims to comprehensively analyze and summarize existing studies on the role of gut microbial GUS in cancer and its interaction with anti-cancer treatments. The goal is to collate and present insights that are directly relevant to patient care and treatment strategies in oncology. By examining the implications of GUS activity in the context of cancer therapy, this review seeks to provide recommendations for clinical practice, highlighting how understanding GUS could potentially enhance treatment efficacy and patient outcomes. Moreover, it aims to identify gaps in current knowledge and suggest directions for future research, thereby contributing to the evolving field of anti-cancer treatment and microbiota research.

## Methods

This scoping review was conducted according to the PRISMA-ScR guidelines(Tricco et al. 2018).

### Eligibility criteria

For this scoping review, we searched for articles from 1990 to 2023 that were written in English and investigated the role of GUS in the gut microbiota in cancer etiologies or anti-cancer treatment. All types of cancer, as were all cancer therapies such as chemotherapy, hormone therapy, and immunotherapy, were included. Only primary research studies performed with humans and rodents were included. Letters, study protocols, case reports, reviews, (conference) abstracts, and studies written in languages other than English were excluded. Studies that investigated the influence of food or nutrition on GUS or studies that did not perform GUS measurements were also excluded. Interventional studies that investigated the effect of a GUSi were included based on the indirect GUS measurements.

### Information sources

The following databases were used in October 2023 to search for potentially relevant articles: PubMed, Embase, and Web of Science. The search strategies were drafted and discussed with an experienced librarian from Maastricht University. The final search strategies can be found in Supplemental Table 1–3. All final search results were exported to Rayyan (Ouzzani et al. 2016).

### Selection of sources of evidence

Two reviewers (LEH and PAJK) independently screened the databases, selecting eligible articles based on their titles and abstracts, while eliminating duplicates. Subsequently, LEH and MAMS independently reviewed the full texts of all potentially relevant articles to determine their eligibility. Any disagreements were resolved through discussion until consensus was achieved. Exclusion reasons were recorded. Furthermore, references of included articles were screened to identify relevant studies not captured in the initial search strategy.

### Data charting process

The data were extracted in duplicate by the two independent reviewers (LEH and MAMS). For each study the following data were retrieved if applicable: first author, year of publication, country, study aim, study design, population (humans or rodents, number of participants, sex, age, and cancer type) and sample size, methods and corresponding outcomes, and results.

## Results

**Fig. 2:**
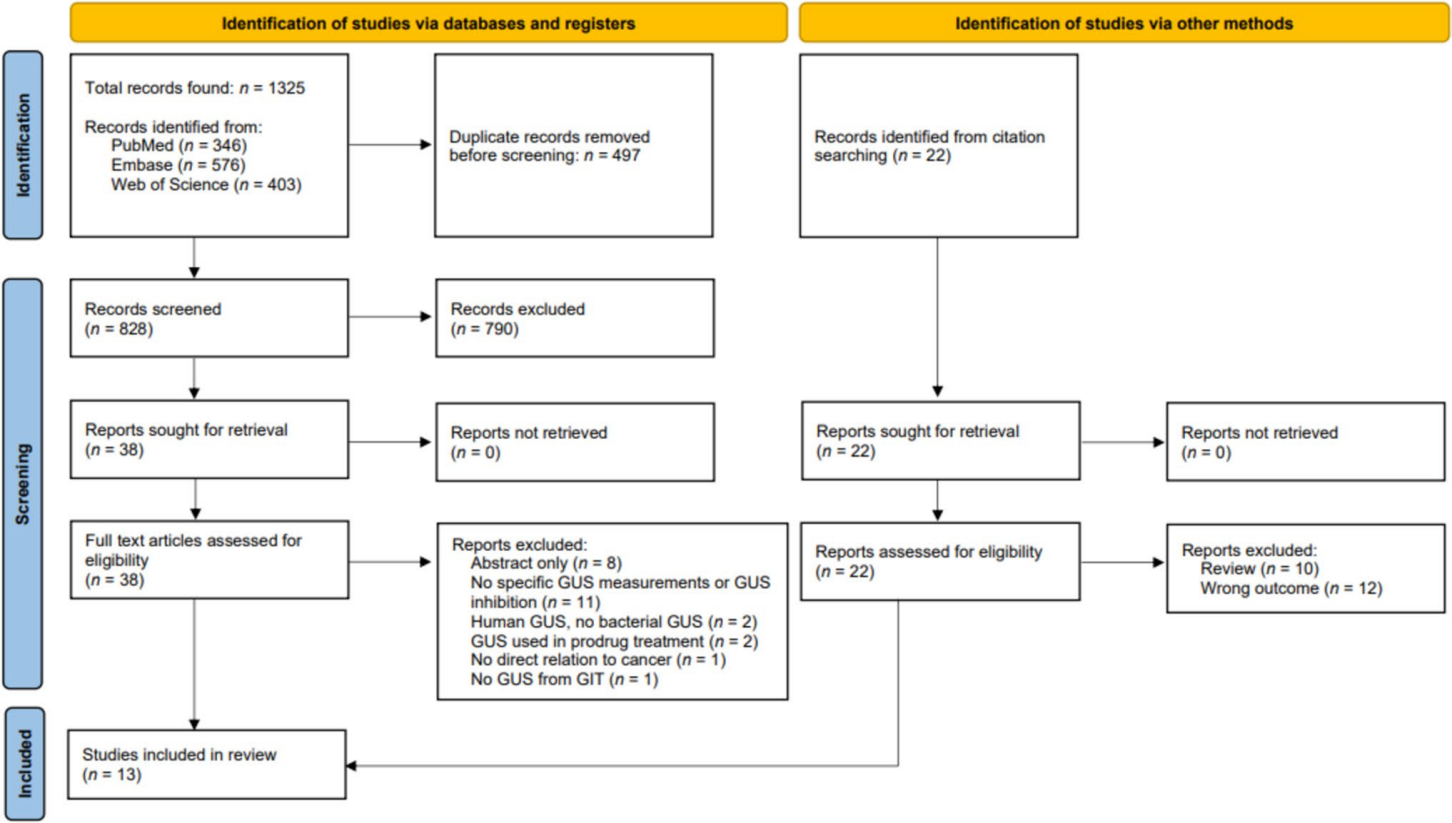
PRISMA 2020 flow diagram for systematic reviews and meta-analyses of the literature search and selection process. GUS: beta-glucuronidases; GIT: gastro-intestinal tract

A total of 1325 studies were identified in the examined databases. After removing duplicates, screening for eligibility, and citation searching, 13 studies were included in the review (Fig. 2). In total three articles investigated the role of GUS in carcinogenesis and ten articles investigated the role of GUS in anti-cancer treatment.

### The role of bacterial GUS in carcinogenesis

In total, three studies investigated GUS in colorectal cancer (CRC) patients and healthy controls, focusing on fecal samples and the genetic makeup of the gut microbiota. No studies were found concerning GUS activity in other types of cancer. Detailed characteristics and results from these studies are summarized in Supplementary Table S4.

Among these studies, only one study performed bacterial GUS activity measurements in CRC patients. Kim and Jin (Kwa et al. 2016) revealed that GUS activity in fecal samples from CRC patients was 1.7 times higher than in healthy controls. Sonication of fecal samples significantly increased GUS activity in both groups, especially in CRC patients (12.1 times) compared to healthy controls (1.8 times). Sonication disrupts cell membranes with the use of sound waves, allowing the extraction of cellular contents, including enzymes. Inhibition of GUS activity using D-saccharic acid 1,4-lactone showed comparable effects in both groups, suggesting the presence of similar GUS types in CRC patients and healthy controls.

In addition to the study above, Li et al. (2015) provided insights into the bacterial composition within fecal samples from CRC patients, revealing an increase in *Escherichia coli* and a decrease in *Lactobacillus spp.* And *Bifidobacterium spp*. Furthermore, PCR amplification and sequencing of a conserved region of microbial GUS from *E. coli* cultured from the fecal samples of CRC patients and healthy controls identified a mutation in the GUS sequence of *E. coli* specific to the CRC group. The mutation involved the change of the 1158th adenine base to a guanine. Whether this mutation encoded for a different amino acid is not clear.

Building on the genetic aspects of GUS activity, Zhang et al. (2019) investigated the GUS activity of gut microbes and the potential of fecal microbiomes to convert PhIP-G. PhIP-G is a representative liver metabolite of 2-amino-1-methyl-6-phenylimidazo [4,5-b] pyridine (PhIP), the most abundant carcinogenic heterocyclic amine in well-cooked meat. GUS from the *Faecalibacterium prausnitzii* was found to efficiently convert PhIP-G to PhIP. Additionally, PhIP hydrolysis positively correlated with GUS activity. The gene abundance of GUS did not differ in fecal samples between investigated CRC patients and healthy individuals. However, the main GUS contributing phyla varied between the two groups. In CRC patients, the proportion of Bacillota (formerly Firmicutes) GUS was lower than that of Bacteroidota (formerly Bacteroides) GUS, while in healthy individuals, the opposite was observed. Since *F. prausnitzii* seemed to be able to convert PhIP-G to PhIP, it might be hypothesized that the proportion of *F. prausnitzii* GUS is higher in CRC patients. However, *F. prausnitzii*, supposed to promote gut health, belongs to the phylum Bacillota, which was not found to be the main GUS contributing phylum in CRC patients compared to healthy controls. This study provided no information about the difference in abundance of *F. prausnitzii* or the proportion of *F. prausnitzii* GUS between CRC and healthy controls.

### The role of bacterial GUS in anti-*cancer* treatment

The effect of irinotecan (also known as CPT-11) on the GI tract and the gut microbiome was investigated by three studies. GUS expression was measured and/or the role of GUS was investigated. Detailed characteristics and results from these studies are summarized in Supplementary Table S5.

In an interventional study by Stringer et al. (2008), rats treated with irinotecan exclusively exhibited early-onset mild and moderate diarrhea (2–48 h after treatment) and late-onset mild diarrhea (72 h after treatment). GUS expression increased in the jejunum and colon post-treatment, while control rats showed consistently low GUS expression. Moreover, in irinotecan-treated rats, GUS-producing bacteria increased and beneficial bacteria (*Lactobacillus spp.* and *Bifidobacterium spp.*) declined. *Lactobacillus spp.* are suggested to inhibit GUS and *Bifidobacterium spp.* seem to have protective properties for the mucosal barrier of the GIT, and are therefore indicated as beneficial bacteria. Irinotecan had no inhibitory effects on tested bacteria. A similar study by the same research group (Stringer et al. 2009) was conducted a year later, extending observations to later time points (96-144 h) post-treatment. Colon GUS expression peaked at 96-120 h, correlating with moderate or severe diarrhea incidence (11% and 6% respectively, at 96 h) and a peak in *E. coli* (96 h).

In a 2015 study (Pedroso et al. 2015), the response to irinotecan, administered as CPT-11, was evaluated in germ-free (GF) and conventional (CV) mice. GF mice were less susceptible to CPT-11 induced intestinal damage compared to CV mice. CV mice injected with CPT-11 had increased inflammatory infiltrates (neutrophils, eosinophils, inflammatory cytokines), decreased height of intestinal villi, increased intestinal permeability, elevated proliferation rate of the intestinal epithelium, and fewer goblet cells in the intestinal mucosa compared to GF mice receiving CPT-11. After CPT-11 injection, GF mice were found to have increased SN-38, the active metabolite of irinotecan, concentrations in the intestinal fluid compared to CV mice. Pedroso et al. attributed this unexpected difference to an earlier finding that SN-38, upon conversion from SN-38G by GUS, is rapidly absorbed by bacterial cell walls or intestinal dietary fiber, resulting in only 10% being measurable in the intestinal fluid. The presence of SN-38 in the intestinal fluid of GF mice, could possibly be explained by the excretion of not only SN-38G but also SN-38 via the bile (Guan et al. 2017). Colonization of GF mice with fecal material from CV mice led to a similar response to CPT-11 as observed in CV mice (changes in intestinal pathology and mucosal inflammation). Investigation into the role of GUS-producing bacteria in irinotecan-induced mucositis revealed that mice administered with *E. coli* harboring the GUS gene showed increased intestinal permeability compared to GUS-lacking *E. coli*. No differences between the two groups were found in the amount of goblet cells, cell proliferation rate, and inflammatory or immune responses. Altogether these results show that treatment with CPT-11 results in increased intestinal damage and permeability when a microbiome is present.

Several studies examined the effect of GUS inhibition on the efficacy and/or side effects of the chemotherapeutic agent irinotecan. A study published in 2010 investigated the ability of a first-generation GUS inhibitor (Inhibitor 1) to alleviate CPT-11 late-onset diarrhea and intestinal damage (Wallace et al. 2010). Only mice receiving CPT-11 experienced (bloody) diarrhea compared to mice that did not receive CPT-11. Inhibitor 1 significantly decreased the number of mice with (bloody) diarrhea. Furthermore, CPT-11 destroyed intestinal glands and the epithelial layer in the mice. This led to an increased presence of inflammatory cells within the lamina propria of the large intestine. When irinotecan was simultaneously administered with Inhibitor 1 the glandular structure of the intestinal tissues was protected.

Subsequently, a study (Wallace et al. 2015) from the same research group assessed the potential of inhibitor R1, the analog of inhibitor 1, to alleviate CPT-11-induced diarrhea in mice with results compared to a control and previously tested inhibitor 1 (Wallace et al. 2010). Co-administration of Inhibitor R1 with CPT-11 reduced the incidence of bloody diarrhea from day 8 to 10 compared to CPT-11 alone, but not as effectively as Inhibitor 1. Plasma levels of CPT-11, SN-38, and SN-38G remained unaffected by Inhibitor 1.

Similarly, Roberts et al. (2013) examined the ability of another GUSi, inhibitor 5, to alleviate CPT-11 induced intestinal toxicity. Mice receiving only CPT-11 showed GI symptoms from day 2 through day 10 after treatment. These GI symptoms included changes in appetite, bowel movements, mobility, and body weight. One-third of the mice in the CPT-11 group exhibited bloody diarrhea on day 8, with all mice displaying this symptom by day 10. However, Inhibitor 5 reduced the incidence of CPT-11 induced diarrhea. No diarrhea was observed on day 8, and only 30% of the mice in this group showed symptoms on day 10. The inhibitor did not affect the average body weight of the mice treated with CPT-11.

Additionally, the impact of the *E. coli* GUSi, TCH-3562, on CPT-11 anti-tumor activity and drug-induced diarrhea was studied in mice with colon carcinoma CT26 cell injections (Cheng et al. 2019). TCH-3562 did not affect plasma levels, peak time, or peak concentration of SN-38 or SN-38G compared to groups receiving CPT-11 alone. However, the area under the curve and half-life of SN-38 and SN-38G increased with TCH-3562 co-administration. The anti-tumor efficacy of CPT-11 remained similar when combined with TCH-3562, with comparable tumor growth observed in both groups. TCH-3562 delayed CPT-11-induced diarrhea onset and reduced recovery duration. Body weight loss was similar between the CPT-11 and CPT-11 + TCH-3562 groups.

Similar results were found in a recent mixed-model study (Bhatt et al. 2020). GUSi (UNC10201652) blocked both the increase of ex vivo fecal GI bacterial GUS activity after 24 h and the reduction of intestinal epithelial cell proliferation five days after irinotecan treatment in FVB mice. GF mice were colonized with wild-type (WT) *E. coli* or GUS gene-deleted *E. coli*. Irinotecan increased ex vivo fecal GUS activity and lipocalin-2 (a marker for gut damage and inflammation) in WT *E. coli* mice more than in the mice colonized with the GUS gene-deleted *E. coli*. Additionally, intestinal cell proliferation was lower in the mice with the GUS gene present in *E. coli*. Furthermore, GUS inhibition protected against weight loss and intestinal toxicity induced by irinotecan treatment (inflammation, crypt damage, and diarrhea) in breast cancer tumor immune-deficient mice. GUSi increased gut microbial diversity and decreased the growth of Pseudomonodota in irinotecan-treated immune-deficient mice. The expansion of Pseudomonodota during irinotecan treatment was assigned to the growth of the family Enterobacteriaceae, which are the only intestinal taxa that encode a GUS operon containing the GUS gene and inner- and outer-membrane glucuronide transporters. In a genetically engineered mouse model (GEMM) bearing breast cancer tumors, GUSi significantly enhanced the antitumor efficacy of irinotecan by protecting the intestines and allowing mice to receive significantly more doses irinotecan. In addition to increasing irinotecan-mediated tumor regression, GUSi also increased survival rates and alleviated GI damage.

Additionally, one study investigated the GUS type that was primarily responsible for the conversion of SN-38G and whether it could be targeted by GUSi. Jariwala et al. (2020) identified and quantified GUS enzymes in human fecal samples using a novel activity-based probe-enabled proteomics pipeline. Among these enzymes, Loop 1 (L1) GUS demonstrated a significant association with the deconjugation of SN-38G and exhibited the highest efficiency in processing SN-38G compared to other GUS variants. Furthermore, piperazine-containing small molecule inhibitors (UNC4917 and UNC10201652) were capable of specifically targeting L1 GUS enzymes. Bacterial species that are known to possess a L1 GUS are *Lactobacillus rhamnosus*, *Ruminococcus gnavus*, *F. prausnitzii*, *E. coli*, *Eubacterium eligens*, *Streptococcus agalactiae*, and *Clostridium perfringens* (Biernat et al. 2019). Despite not being the most abundant type found in the individual human microbiome, L1 GUS was present in approximately two-thirds of the individuals studied by Pollet et al. (2017).

Beyond irinotecan, the effects of gut microbial GUS enzymes on the reactivation of another anti-cancer therapeutic known to be glucuronidated and cause gut toxicity, the selective tyrosine kinase inhibitor regorafenib, has been investigated. Ervin et al. (2019a) measured the conversion of regorafenib-glucuronide to regorafenib in murine and human fecal samples. No conversion of regorafenib-glucuronide was detected over four hours in either murine or human fecal samples. Subsequent experiments on fecal samples from three specific pathogen-free (SPF) mice revealed active GUS throughout the GI tract, with complete conversion of regorafenib-glucuronide to regorafenib observed by 48 h. Ex vivo fecal lysate experiments in five GF mice did not show any conversion into regorafenib, suggesting the necessity of gut microbial enzymes for this conversion. Three recently formulated GUSi (UNC7084, UNC7087, and UNC7159) successfully suppressed the deconjugation process of regorafenib-glucuronide in cecal mixture samples from two out of three SPF mice. Among these inhibitors, two effectively inhibited deconjugation in all three SPF mice, while one (UNC7084) failed to inhibit deconjugation in the cecal mixture sample of one SPF mouse. Furthermore, a panel of diverse purified gut microbial GUS enzymes was examined for regorafenib-glucuronide processing in vitro, and revealed that only a small number of unique Flavin mononucleotide (FMN)-binding GUS enzymes performed this reaction. FMN GUS enzymes are a recently discovered set of microbial isoforms and have been shown to process both small and large glucuronidated substrates, including drug-like compounds (Pellock et al. 2019; Zhang et al. 2022). A possible explanation for the activities of only FMN GUS in processing regorafenib-glucuronide is that this substrate contains its glucuronide uniquely appended to a nitrogen atom in the center of the regorafenib molecule, while most other drug glucuronides are oxygen-linked and in a more terminal position in the parent drug. Thus, distinct substrates are processed by discreet gut microbial GUS isoforms, suggesting that future investigations may facilitate personalized approaches to treatment outcomes in human patients.

## Discussion

This scoping review aimed to comprehensively analyze and summarize existing studies on the roles of gut microbial GUS enzymes in cancer and anti-cancer treatments with the goal of collating and presenting insights directly relevant to patient care and treatment strategies in oncology. Across the studies in this review, variations in GUS activity and GUS genetic sequences associated with CRC highlight the enzyme's potential role in carcinogenesis, but more research is required. Other studies also observed increased *E. coli* and decreased *Bifidobacterium spp.* in CRC patients (Chen et al. 2012; Liu et al. 2021). In particular, *E. coli*, a GUS-producing microbe supposed to contribute to the degradation of the intestinal mucosal barrier, is of special interest in CRC (Dashnyam et al. 2018; Kwa et al. 2016; Quaglio et al. 2022). Additional genera, such as Lactobacilli and Bifidobacteria, are thought to provide protection by competing for adhesion sites in the GI tract, thereby guarding against potential carcinogenesis-promoting pathogens (Tortora et al. 2022). The observed differences in microbial phyla contributing to GUS activity between CRC patients and healthy controls may offer possibilities for further research into microbial influences on carcinogenesis.

Research on gut microbial GUS activity has primarily focused on CRC, with observational studies investigating its role in other types of cancer being scarce or absent. A potential role of GUS has been identified in relation to breast cancer and ovarian cancer, suggested by its link to estrogen metabolism and therefore estrogen-related diseases. Estrogen glucuronides, which are excreted via bile for fecal elimination, are deconjugated and thereby reactivated in the intestines by bacterial GUS. Through enterohepatic circulation, these reactivated estrogens re-enter the systemic circulation. By attaching to estrogen receptors on breast cancer cells, the reactivated estrogens may promote the progression of estrogen-driven breast and ovarian cancer (Hu et al. 2023; Kwa et al. 2016; Sui et al. 2021). Additionally, estrogens play a role in the development of breast as well as other cancers. Quinone adducts and reactive oxygen species released during estrogen metabolism can increase DNA mutations, leading to carcinogenesis (Wen et al. 2017; Yager & Davidson 2006). Studies have demonstrated that higher serum endogenous estrogen concentrations in postmenopausal women significantly increase the relative risk of breast cancer compared to those with lower serum concentrations (Key et al. 2002). Despite the strong indications of the involvement of gut microbial GUS in breast and ovarian cancer, there is a notable lack of observational studies measuring GUS activity in these patients.

Studies have also explored how gut microbial GUS enzymes affect the transformation of the cancer drugs irinotecan and regorafenib from their inactive forms into active ones in rodents. This process is crucial for drug efficacy and toxicity. Combined findings suggest that irinotecan treatment in rodents induces early and late-onset diarrhea, accompanied by increased GUS expression in the jejunum and colon, with shifts in microbial composition favoring GUS-producing strains. Furthermore, irinotecan treatment led to damage to the intestinal barrier resulting in increased permeability and inflammatory infiltrates. Additionally, GF mice demonstrate reduced susceptibility to irinotecan-induced intestinal damage compared to CV mice, which exhibit inflammatory responses and decreased intestinal integrity after irinotecan treatment. Colonization of GF mice with CV microbiota reproduces the adverse effects observed in CV mice. Furthermore, *E. coli* with an intact GUS gene, but not GUS-minus *E. coli*, contributes to increased intestinal permeability post-irinotecan treatment.

The collective findings from several studies provide insight into how inhibiting GUS affects both the efficacy and toxicity of the chemotherapeutic agent irinotecan in mice. Research has explored the use of different GUSi in mitigating irinotecan-induced toxicity. Inhibitor 1 showed promise in protecting intestinal tissues and reducing the incidence of diarrhea. While Inhibitor R1 showed some efficacy, it was not as effective as Inhibitor 1 in reducing diarrhea incidence. Plasma levels of irinotecan metabolites remained unaffected by Inhibitor 1. Another GUSi, Inhibitor 5, demonstrated potential in reducing CPT-11 induced GI symptoms in mice. It effectively decreased the incidence of diarrhea without affecting body weight. Similarly, *E. coli* GUSi TCH-3562 delayed the onset of CPT-11 induced diarrhea and improved recovery duration without affecting anti-tumor efficacy of the treatment or plasma levels of irinotecan metabolites in mice with colon carcinoma. GUSi UNC10201652 demonstrated efficacy in blocking intestinal damage and inflammation induced by irinotecan treatment. In breast cancer GEMM mice, it also increased gut microbial diversity and reduced the growth of Proteobacteria. Additionally, anti-tumor efficacy and survival rates increased when mice treated with irinotecan were co-administred with the GUSi.

Additionally, one study found that L1 GUS was primarily responsible for the conversion of SN-38G to SN-38. Inhibitors tested in this study showed that they were able to target L1 GUS specifically. Previous studies made a distinction between different subtypes of GUS: L1, Mini-Loop 1, Loop 2, Mini-Loop 2, Mini-Loop 1,2, N-terminal loop, FMN-binding, and No Loop GUSs (Pellock et al. 2019; Pollet et al. 2017; Walker et al. 2022). The subtypes demonstrate distinct functionality, where longer loops (L1, Mini-Loop 1, Loop 2) can effectively process small glucuronide substrates, and subtypes with an open active site cleave a larger heparan substrate (Pollet et al. 2017). While L1 has been identified as primarily responsible for the conversion of SN-38G, it is possible that other subtypes of GUS could be involved in the metabolism of other (anti-cancer) drugs. Complete conversion of regorafenib-glucuronide was observed in SPF mice within 48 h, indicating mediation by gut microbial enzymes. Testing of three GUSi showed suppression of conversion in SPF mice, except for one inhibitor in a single sample, suggesting the presence of a potential undiscovered GUS variant. This study further showed that a small set of FMN GUS were responsible for regorafenib-glucuronide processing in vitro.

Overall, blocking the actions of gut microbial GUS enzymes in rodent studies have provided several lines of evidence to show this approach can reduce the harmful side effects associated with the cancer drug irinotecan. Interestingly, this approach does not compromise the drug's ability to fight tumors and has been shown to increase anti-tumor efficacy by preventing dose-limiting gut toxicity (Bhatt et al. 2020). These findings highlight the potential of GUSi as an adjunct therapy in anti-cancer treatment to improve patient outcomes and reduce toxicity.

Furthermore, Elmassry et al. have identified 11 antineoplastic agents, including irinotecan and regorafenib, which are potentially affected by GUS since their metabolisms involve glucuronidation (Elmassry et al. 2021). Other described agents were axitinib, enasidenib, epirubicin, erlotinib, etoposide, rucaparib, sorafenib, tamoxifen, and vadimezan. The role of gut microbial GUS in sorafenib-glucuronide conversion was proposed in 2015 because sorafenib-glucuronide levels decreased while sorafenib levels increased over time in the cecal contents of mice (Vasilyeva et al. 2015). Heat treatment halted sorafenib formation and pretreatment with neomycin to reduce gut flora decreased the conversion of sorafenib-glucuronide to sorafenib, pointing to a gut microbial enzyme-mediated deconjugation process. Indeed, in vivo studies in mice showed that sorafenib-glucuronide biliary excretion led to prolonged plasma levels of reactivated sorafenib, observations that would require intestinal removal of the inactivating glucuronide component, possibly by GUS.

The role of GUS in the conversion and potential side effects of sorafenib and other antineoplastic agents warrants further investigation in the future. Irinotecan and regorafenib are commonly used to treat solid tumors. Irinotecan is administered as monotherapy or in combination therapies to CRC patients, and is also part of regimens addressing pancreatic and lung cancers (de Man et al. 2018; Kciuk et al. 2020). Regorafenib is employed to treat CRC, hepatocellular carcinoma, and GI stromal tumors (GIST) (Grothey et al. 2020). Patients undergoing treatment with irinotecan or regorafenib may potentially benefit from a GUSi. If GUS inhibition in healthy humans and cancer patients ultimately proves to be safe and equally or more effective in reducing irinotecan-induced toxicity without compromising treatment efficacy, as shown in mice, GUSi during irinotecan therapy could potentially become standard practice. This applies similarly to regorafenib and other anti-cancer therapies that reach the gut as glucuronides and are known to cause GI toxicity. However, further research is required in this area.

Besides the potential role of GUSi in anti-cancer treatment, more strategies modifying gut microbial GUS could be developed. For example, CRISPR-Cas systems can control gene expression and modulate the production of metabolites and proteins (Ramachandran and Bikard 2019). Adapted CRISPR-Cas systems could therefore potentially modify GUS gene expression and GUS activity. Additionally, gut microbial GUS activity could also be altered by transferring a whole gut microbial community via fecal microbiota transplantation (Biazzo and Deidda 2022), or by supplementation of pre- or probiotics. A previous study already showed a reduction in GUS activity after four weeks of pre- or probiotic supplementation in humans (De Preter et al. 2008).

To the best of our knowledge, this is the first review to comprehensively analyze and summarize the existing studies on the role of gut microbial GUS enzymes in cancer, as well as the interactions of these non-human proteins with chemotherapeutic agents. We sought to offer insights directly relevant to patient care and treatment strategies in the field of oncology. By employing a broad scope and a thorough, systematic search across multiple databases, we found evidence that gut microbial GUS has the potential to influence both cancer etiology and the efficacy of anti-cancer treatments, and we identified gaps in the literature that provide valuable directions for future investigations. However, this review also has limitations. Given the nature of the scoping review approach, all published literature was included without regard to study quality. The findings presented in this review are predominantly based on rodent studies, with cross-sectional studies in cancer patients being scarce. To our knowledge, there is currently one active study exploring GUS activity in CRC patients undergoing systemic treatment with irinotecan (NCT05655780) (US National Library of Medicine 2023). Interest in gut microbial GUS enzymes is growing and further research is highly recommended to enhance our knowledge and implementation in the field of oncology.

## Conclusion

This scoping review has provided insights into the complex role of gut microbial GUS in carcinogenesis and its interaction with anti-cancer agents, notably irinotecan. While the precise role of GUS in cancer development needs more exploration, the review has shown potential paths for the future application of GUSi to mitigate side effects and possibly enhance the efficacy of anti-cancer treatments. Moreover, the observed effects of GUS inhibition in rodent models, including reduced toxicity and maintained or improved antitumor efficacy of chemotherapeutic agents, highlight the promising potential of GUSi in improving patient outcomes. It can be seen as an initial step towards future therapeutic options. Despite these promising findings, the review acknowledges the scarcity of cross-sectional studies on GUS activity in cancer patients, identifying this as a critical gap in the current literature. As such, it advocates for further research into GUS inhibitors and their clinical applications, with a strong call for more observational studies to elucidate GUS's role in carcinogenesis and its potential as a target in anti-cancer treatment strategies. This will not only deepen our understanding of GUS's biological functions but also open the door for innovative approaches to enhance the effectiveness and safety of cancer therapies.

## Supplementary Information

Below is the link to the electronic supplementary material.

Supplementary file1 (PDF 341 KB)

## Data Availability

No datasets were generated or analysed during the current study.
