# Supplementary material for: The role of gut microbial β-glucuronidases in carcinogenesis and cancer treatment: a scoping review
Source: J Cancer Res Clin Oncol. 2024 Nov 13;150(11):495. doi: 10.1007/s00432-024-06028-2 (PMC11561038; doi:10.1007/s00432-024-06028-2)
Supplement: Supplementary file 1 — Supplementary file1 (PDF 341 KB) [file 432_2024_6028_MOESM1_ESM.pdf]

Supplementary materials

Table S1: Search strategies Pubmed

	Search terms	Results
1	"Microbiota"[MeSH Terms] OR "Bacteria"[MeSH Terms] OR "Microbiota"[Title/Abstract] OR "microbiome"[Title/Abstract] OR "microflora"[Title/Abstract] OR "flora"[Title/Abstract] OR "Bacteria"[Title/Abstract] OR "microbial communit*"[Title/Abstract]	1.777.085
2	"Glucuronidase"[MeSH Terms] OR "endo beta d glucuron*"[Title/Abstract] OR "beta glucuron*"[Title/Abstract] OR "endoglucuron*"[Title/Abstract] OR "exo beta d glucuron*"[Title/Abstract] OR "Glucuronidase"[Title/Abstract] OR "GUS"[Title/Abstract] OR "Estrobolome"[Title/Abstract]	26265
3	"Carcinogenesis"[MeSH Terms] OR "Neoplasms"[MeSH Terms] OR "oncogenes*"[Title/Abstract] OR "tumorigenes*"[Title/Abstract] OR "carcinogenes*"[Title/Abstract] OR "tumo*"[Title/Abstract] OR "neoplas*"[Title/Abstract] OR "malignan*"[Title/Abstract] OR "cancer*"[Title/Abstract] OR "antineoplastic agents"[MeSH Terms] OR "anticancer*"[Title/Abstract] OR "antineoplastic*"[Title/Abstract] OR "antitumor*"[Title/Abstract] OR "cancer chemotherap*"[Title/Abstract] OR "chemotherapeutic anticancer*"[Title/Abstract]	5058456
1 and 2 and 3	("Microbiota"[MeSH Terms] OR "Bacteria"[MeSH Terms] OR "Microbiota"[Title/Abstract] OR "microbiome"[Title/Abstract] OR "microflora"[Title/Abstract] OR "flora"[Title/Abstract] OR "Bacteria"[Title/Abstract] OR "microbial communit*"[Title/Abstract]) AND ("Glucuronidase"[MeSH Terms] OR "endo beta d glucuron*"[Title/Abstract] OR "beta glucuron*"[Title/Abstract] OR "endoglucuron*"[Title/Abstract] OR "exo beta d glucuron*"[Title/Abstract] OR "Glucuronidase"[Title/Abstract] OR "GUS"[Title/Abstract] OR "Estrobolome"[Title/Abstract]) AND ("Carcinogenesis"[MeSH Terms] OR "Neoplasms"[MeSH Terms] OR "oncogenes*"[Title/Abstract] OR "tumorigenes*"[Title/Abstract] OR "carcinogenes*"[Title/Abstract] OR "tumo*"[Title/Abstract] OR "neoplas*"[Title/Abstract] OR "malignan*"[Title/Abstract] OR "cancer*"[Title/Abstract] OR "antineoplastic agents"[MeSH Terms] OR "anticancer*"[Title/Abstract] OR "antineoplastic*"[Title/Abstract] OR "antitumor*"[Title/Abstract] OR "cancer chemotherap*"[Title/Abstract] OR "chemotherapeutic anticancer*"[Title/Abstract])	**346**

Table S2: Search strategies Embase

	Search terms	Results
1	exp microflora/ OR exp microbiome/ OR exp flora/ OR exp bacterium/ OR exp microbial community/ OR (microflora OR microbiota OR bacteria OR microbiome OR flora OR microbial communit* OR Bacterium).ti,ab,kf.	2.129.678
2	Exp beta glucuronidase/ OR (glucuronidase OR endo beta d glucuron* OR beta glucuron* OR endoglucuron* OR exo beta d glucuron* OR GUS OR Estrobolome).ti,ab,kf.	21.928
3	exp carcinogenesis/ OR exp neoplasm/ OR exp malignant neoplasm/ OR exp antineoplastic agent/ OR exp cancer chemotherapy/ OR (oncogenes* OR tumorigenes* OR carcinogenes* OR tumo* OR neoplas* OR malignan* OR cancer* OR antineoplastic* OR anticancer* OR antitumor* OR cancer chemotherap* OR chemotherapeutic anticancer*).ti,ab,kf.	7.802.188
1 and 2 and 3	(exp microflora/ OR exp microbiome/ OR exp flora/ OR exp bacterium/ OR exp microbial community/ OR (microflora OR microbiota OR bacteria OR microbiome OR flora OR microbial communit* OR Bacterium).ti,ab,kf.) AND (Exp beta glucuronidase/ OR (glucuronidase OR endo beta d glucuron* OR beta glucuron* OR endoglucuron* OR exo beta d glucuron* OR GUS OR Estrobolome).ti,ab,kf.) AND (exp carcinogenesis/ OR exp neoplasm/ OR exp malignant	**576**

	neoplasm/ OR exp antineoplastic agent/ OR exp cancer chemotherapy/ OR (oncogenes* OR tumorigenes* OR carcinogenes* OR tumo* OR neoplas* OR malignan* OR cancer* OR antineoplastic* OR anticancer* OR antitumor* OR cancer chemotherap* OR chemotherapeutic anticancer*).ti,ab,kf.)	

Table S3: Search strategies Web of Science

	**Search terms**	**Results**
1	(TS=(Microbiota OR microbiome OR microflora OR flora OR bacteria OR microbial communit*))	950,635
2	(TS=("endo beta d glucuron*" OR "beta glucuron*" OR "endoglucuron*" OR "exo beta d glucuron*" OR "Glucuronidase" OR "GUS" OR "Estrobolome"))	19,623
3	((TS=("oncogenes*" OR "tumorigenes*" OR "carcinogenes*" OR "tumo*" OR "neoplas*" OR "malignan*" OR "cancer*")) OR (TS=("anticancer*" OR "antineoplastic*" OR "antitumor*" OR "cancer chemotherap*" OR "chemotherapeutic anticancer*")))	4,289,717
1 and 2 and 3	#1 AND #2 AND #3	**403**

Table S4: Description and main findings of studies investigating GUS and carcinogenesis

Author	Year	Country	Study aim	Study design	Population and sample size	Methods and outcomes	Results
Kim et al.(1)	2001	Korea	Investigate the intestinal bacterial GUS activity of colon cancer patients and healthy controls and the factors inducing GUS activity.	Cross-sectional, exploratory study	13 CRC patients (n=8 males, n=5 females) and 5 healthy controls (n=3 males, n=2 females). Patients were Healthy controls aged 40-60 years and healthy controls 20-60 years old.<br><br>Exclusion criteria: current medication, particularly regular or current use of antibiotics	GUS enzyme activity assay in fecal lysate using p-nitrophenyl-β-D-glucuronidase (PNPG) as substrate<br><br>Two fecal lysate preparations:<br>1. Fecal suspension (fecal sample + saline) centrifuged, supernatant used for determination of enzyme activity<br>2. Fecal suspension (fecal sample + saline) centrifuged, sonicated, centrifuged, used for determination of enzyme activity<br><br>Dry weight of fecal samples<br><br>Inhibitory activity of D-saccharic acid 1,4-lactone on the GUS activities in CRC patients (n=3) and healthy control (n=1) was measured	1. Fecal GUS activity CRC patients 1.7x ↑ than in healthy controls<br><br>2. Fecal GUS activity of healthy controls ↑ 1.8x, and the GUS activity of CRC patients ↑ 12.1x.<br><br>Dry-weight fecal samples<br>- Healthy controls 2.6 umol/min/g<br>- CRC patients 30 umol/min/g<br><br>Fecal GUS activity of CRC patients and healthy controls was similarly inhibited by D-saccharic acid 1,4-lactone.
Li et al.(2)	2015	China	1. Analyze intestinal microecology differences between CRC patients and healthy controls<br><br>2. Explore intestinal flora in CRC patients	Cross-sectional, exploratory study	30 CRC patients and 10 healthy controls<br><br>Exclusion criteria: antibiotics, viable microecological agents, stimulant laxatives, polyethylene glycol, or other cathartic preparations one month before sampling<br>No information is available about baseline characteristics.	Fecal samples were cultured and total bacterial genomic DNA and cultured *Escherichia coli* DNA were extracted from feces.<br><br>Genomic DNA and *E. coli* DNA were amplified by PCR and sequenced. Sequences were compared to uidA sequence encoding GUS.	Intestinal flora CRC vs healthy controls<br>- Amount of *E. coli* ↑ significantly<br>- *Lactobacillus spp.* and *Bifidobacterium spp.* reduced significantly<br>- Proportional quantity of anaerobic bacteria and aerobic bacteria reversed<br><br>The intestinal flora carry GUS in both groups<br>Homologies with uidA gene sequences encoding the GUS were 99% (healthy) and 98% (CRC) respectively. In both groups, the 1141th and

			3. Investigate the link between intestinal flora-produced GUS and CRC<br><br>4. Compare GUS sequences from *E. Coli* between the two groups			Specific primers for the GUS gene (S69414.1) were designed and amplified by PCR (as templates of fecal bacteria genomic DNA and *E. coli* DNA resp.)	1148th A bases were deleted, and the 1149th A base was muted into the T base. For the CRC group, the 1158th A base was muted into the G base.
Zhang et al.(3)	2019	Switzerland	Investigate GDH and GUS activity of gut microbes in single and co-cultures<br><br>Investigate the potential of fecal microbiomes to convert PhIP-G to PhIP-M1	A. Experimental<br><br>B. Cross-sectional, exploratory study	A. Representative strains of species predicted to possess GDH activity and gut microbes harboring GUS were tested for GDH and GUS activity.<br><br>B. Metagenomes of 53 CRC state I-IV patients and 103 healthy individuals from a French cohort.	Multidisciplinary approach to investigate microbial activities<br>- Bacterial cultivation of various strains under specific conditions.<br>-Analysis of substrate consumption and metabolite formation using HPLC-RI<br>- GUS activity evaluation in fecal lysate using PNP-G as substrate.<br>- DNA isolation and qPCR from cultures.<br>- Advanced compound analysis using nanoLC-ESI-MS^2<br>- Quantifying gene abundance in fecal metagenomes for GUS and GDH activities	A.<br>- GDH of *Flavonifractor plautii, Blautia obeum, Eubacterium hallii*, and *Lactobacillus reuteri* converted PhIP to PhIP-M1 in the presence of glycerol<br>- GUS of *Faecalibacterium prausnitzii* converted PhIP-G to PhIP<br>- GUS- and GDH-positive bacteria cooperatively converted PhIP-G to PhIP-M1<br>- PhIP-G hydrolysis linearly and positively correlated with GUS activity (plateau +- 1.0 U/mg protein)<br><br>B.<br>- Mean gene abundance of GUS did not differ between healthy donors and CRC patients<br>- Proportions of main contributing phyla were significantly different between the two groups. Firmicutes ↑ and Bacteroidetes ↓ in healthy donors compared to CRC patients.<br>- CRC patients ↓ in abundance of taxa with confirmed GDH and HCA transformation activity

GUS: β-glucuronidase; CRC: colorectal cancer; PhIP: 2-Amino-1-methyl-6-phenylimidazo [4,5-b] pyridine; PhIP-G: PhIP-N2-β-D-glucuronide; HCA: heterocyclic amines

Table S5: Description and main findings of studies investigating GUS and cancer treatment

Author	Year	Country	Study aim	Study design	Population and sample size	Methods and outcomes	Results
Stringer AM et al.(4)	2008	Australia	1. Quantify bacteria known to produce or inhibit GUS at various intervals after treatment with irinotecan<br>2. Investigate the expression of GUS in the GIT<br>3. Determine if bacteria known to colonize the GIT are susceptible to irinotecan	Controlled experimental study using animal subjects	81 healthy female DA rats, 150-175g, housed under controlled conditions in 9 groups (n=9 per group) based on time points (30, 60, and 90 min, 2, 6, 12, 24, 48, and 72h). Each group had an intervention group (n=6) receiving irinotecan treatment (200mg/kg) and a control group (n=3) receiving no treatment	1. Diarrhea incidence and severity was graded (grades 0-3)<br><br>2. Stomach, Jejunum villi and crypts, and colon crypts were incubated with primary antibody (anti-*E. coli* GUS). Expression of GUS was measured in cytoplasm and enterocytes (grades 0-4).<br><br>3. Quantification of specific bacteria using real-time PCR with primers for *Bacteroides spp., Bifidobacterium spp., Clostridium spp., E. coli, Lactobacillus spp.,* and *Staphylococcus spp*.<br><br>4. Bacterial susceptibility to irinotecan using a standard antibiotic susceptibility testing method	1. Diarrhea<br>Treated rats:<br>- Mild diarrhea: 2h 23%, 2-6h 23%, 12h 30%, 24h 39%, 48h 20%, 72h 33%<br>- Moderate diarrhea: 12h 5%, 24h 12%<br>Control rats:<br>No diarrhea at any investigated time point<br><br>2. Immunohistochemistry<br>Treated rats:<br>- Stomach, GUS expression did not alter after irinotecan<br>Jejenum villi, GUS expression ↑ at 2h, largest ↑ at 72h<br>Jejunum crypts, ↑ GUS expression at 24h, 72h<br>- Colon crypts, considerable ↑ GUS expression at 72h<br><br>Control rats:<br>Consistently low GUS expression level<br><br>3. Real-time PCR<br>GUS producing bacteria<br>- *Bacteroides spp.* ↓ 6-24h, and 72h<br>- *Staphylococcus spp.* ↑ 2-12h<br>- *Clostridium spp.* ↑ at 48h<br>- *E. coli* ↑ from 24-48h<br>Beneficial bacteria<br>- *Lactobacillus spp.* ↓ 12-48h<br>- *Bifidobacterium spp.* ↓ at all timepoints after irinotecan treatment<br><br>4. Bacterial susceptibility to irinotecan<br>Irinotecan had no inhibitory effects on the tested bacteria

Stringer AM et al.(5)	2009	Australia	Determine if the early changes seen in the luminal environment, specifically changes in GI flora and intestinal mucins, were also observed during irinotecan-induced late-onset diarrhea in rats treated with irinotecan.	Controlled experimental study using animal subjects	27 healthy DA rats, 150-175g, housed under controlled conditions in 3 groups based on time points (96h, 120h, and 144h). Each group had an intervention group (n=6) receiving irinotecan treatment (200mg/kg) and a control group (n=3) receiving no treatment.	1. Diarrhea was graded (grades 0-3) and body weight was measured<br><br>2. Changes in the luminal environment were measured<br>a. Blood serum samples (sodium, potassium, bicarbonate, chloride, anion gap, osmolality)<br>b. Stomach, small intestine, and colon dissected for histology and staining. Sections were stained with Alcian Blue-PAS, analyzing the number of goblet cells and percentage of cavitated cells, and incubated with primary antibody (Anti-MUC (1-4), Anti-KLF 4, Anti-GUS).<br>c. Culturing bacteria (*Clostridium spp., Escherichia spp, Serratia spp., Enterococcus spp.,* and *Staphylococcus spp*.) and quantification of specific bacteria using real-time PCR with primers (*Bacteroides spp., Bifidobacterium spp., Clostridium spp., E. coli, Lactobacillus spp.,* and *Staphylococcus spp.)*	1. Diarrhea and body weight<br>Treated rats:<br>- Mild diarrhea: 0-24h 23%, 48h 6%, 72h 28%, 96h 33%<br>- Moderate diarrhea: 72h 5%, 96h 11%<br>- Severe diarrhea: 72h 6%, 96h 6%<br>Weight decreased until 96h, increased weight from 120h on.<br>Control rats:<br>no diarrhea at any investigated time point<br>Gained weight gradually and consistently.<br><br>2. Changes in luminal environment<br>a. Electrolytes<br>Irinotecan-treated rats vs control: sodium and bicarbonate ↓ (96h), potassium ↑ (AT), anion gap ↑ (96h), osmolality ↓ (AT)<br>b. Histology<br>Pathological changes were seen in the jejunum and colon. The jejunum showed ↑ mitotic activity (96h), and occasional apoptotic bodies (96-144h). The colon showed considerable damage (96-120h)<br><br>Goblet cells:<br>In the jejunum total goblet cells ↑, no difference in intact goblet cells, cavitated goblet cells ↑<br>In the colon total goblet cells ↓ (96h), returned to control levels (144h), intact goblet cells ↓ (96h), and no difference in cavitated goblet cells (%↑ at 96h)<br><br>MUC gene expression:<br>- MUC 1 and MUC 2 expression did not alter significantly in the jejunum or colon<br>- MUC 3 no significant changes jejunum (AT), not expressed in colon<br>- MUC 4 expression was significantly ↓ at 96h in the jejunum villi<br>- KLF 4-expressing cells were significantly ↓ in the jejunum villi at 96h and 144h

							- Colon GUS expression was ↑ 96h-120h after treatment<br><br>c. Cultured bacteria:<br>In the jejunum ↑ *Escherichia spp*. a peak at 96h, ↑ *Clostridium spp*. 96h-144h, and ↑ *Staphylococcus spp*. peak at 144h. In the colon ↑ *Escherichia spp*. 96h-144h, small ↑ *Clostridium spp.* at 96h, ↑ *Enterococcus spp.* 120h-144h, ↑ *Serratia spp.* peak at 120h, and ↑ *Staphylococcus spp*. at 120h-144h<br><br>Quantification of fecal flora:<br>No significant changes were found. *E. coli* peak at 96h and ↓ *Bifidobacterium spp*. after treatment (AT).<br><br>↑ GUS expression was correlated with the incidence of severe diarrhea and a peak in *E. coli*

Wallace BD et al.(6)	2010	USA	Develop potent inhibitors of bacterial GUS enzymes without harming essential commensal bacteria or mammalian cells<br><br>*Sub aim:* Examine the ability of Inhibitor 1 to eliminate the delayed diarrhea and intestinal damage caused by CPT-11 administration	Controlled experimental study using animal subjects	64 healthy female Balb/cJ 6- to 8-week-old mice, housed in controlled conditions in 4 groups (n=16 per group):<br>- Group 1: 50 µl ddH2O, i.p. + 1% DMSO, oral gavage, twice daily<br>- Group 2: 10 µg Inhibitor 1, oral gavage, twice daily<br>- Group 3: CPT-11 i.p. (50mg/kg of body weight), once daily for 9 days<br>- Group 4: CPT-11 + Inhibitor 1 (dose and schedule identical to group 2 and 3, resp.)	1. Daily monitoring of body weight, stool consistency, occult blood in stool<br><br>2. Dissection of large intestines and H&E staining for examination and scoring of colonic tissue samples:<br>- Cell infiltration of inflammatory cells was counted and scored<br>- Epithelial damage was scored	1. GI symptoms appeared after 7 days<br>Group 1 & 2: no diarrhea at AT during the study<br>Group 3: On days 8-10 all mice had diarrhea and bloody stool, day 11 all mice were euthanized<br>Group 4: less diarrhea and less bloody stool compared to group 3<br><br>2. Group 1 & 2: healthy glandular structure and intact epithelial layer<br>Group 3: CPT-11 administration destroyed glandular structure and epithelial layer, eliminating the glands and causing a large influx of inflammatory cells<br>Group 4: Inhibitor 1 protected the mice GIT epithelium from CPT-11–induced damage
Roberts AB et al.(7)	2013	USA	Characterize novel microbial GUS inhibitors that selectively target *E. coli* GUS.<br><br>*Sub aim:* To examine the ability of Inhibitor 5 to alleviate CPT-11-induced intestinal toxicity in mice	Controlled experimental study using animal subjects	36 healthy female Balb/cJ 8-10 weeks old mice, housed in controlled conditions in 4 groups (n=9 per group):<br>- Group 1: vehicle controls<br>An equivalent volume of 0.25% (w/v) carboxymethylcellulose sodium salt solution i.p. and by oral gavage (~100 ml 2x/day)<br>- Group 2: Inhibitor 5<br>Inhibitor 5 gavaged (10 µg/d) 2x/day (every 10 hours) starting on day 1 and oral gavage of 0.25% (w/v) carboxymethylcellulose sodium salt solution and i.p. 1x/day<br>- Group 3: CPT-11<br>CPT-11 injected (50 mg/kg) i.p. 1x/day in the morning with oral gavage of 0.25% (w/v) carboxymethylcellulose sodium	Daily monitoring of body weight, stool consistency, occult blood in stool, and GI symptoms (appetite, bowel movements, mobility, etc.)	Groups 1 & 2: no evidence of toxicity throughout the experiment. No changes in body weight.<br>Group 3: On day 2 GI symptoms were observed. Day 8 bloody stool in one-third of the mice, all mice by day 10<br>Group 4: No bloody diarrhea by day 8, 30% showed symptoms by day 10.<br><br>Inhibitor 5 significantly ↓ the incidence of acute GI toxicity caused by CPT-11.

					salt solution<br>- Group 4: CPT-11 and Inhibitor 5 CPT-11 injected (50 mg/kg) i.p. 1x/day in the morning and Inhibitor 5 gavaged (10 µg/d) 2x/day (every 10 hours).		

Pedroso SHSP et al.(8)	2015	Brazil	Evaluate the differences in response to irinotecan treatment between GF and CV mice, specifically focusing on the role of GUS-producing bacteria in the development of irinotecan-induced mucositis.	Controlled experimental study using animal subjects	100 healthy female NIH Swiss 6-8-week-old GF and CV mice, housed in controlled conditions in 10 groups (n=10 per group, n=4 histological analysis, n=6 other parameters):<br><br><u>Group 1</u>: CV control<br>Received i.p. saline<br><u>Group 2</u>: GF control<br>Received i.p. saline<br><u>Group 3</u>: CVO control Received orally fresh diluted feces of CV and i.p. saline<br><u>Group 4</u>: CV+CPT-11<br>Received i.p. irinotecan<br><u>Group 5</u>: GF+CPT-11<br>Received i.p. irinotecan<br><u>Group 6</u>: CVO+CPT-11 =<br>Received orally fresh diluted feces of CV and i.p. irinotecan<br><u>Group 7</u>: MN-TG1 Gnotobiotic mice orally mono-associated with *E. coli* producing GUS<br><u>Group 8</u>: MN-TG1+CPT-11 Same as group 7 but with i.p. irinotecan<br><u>Group 9</u>: MN-L91<br>Gnotobiotic mice orally mono-associated with *E. coli* strain deleted for the gene encoding GUS<br><u>Group 10</u>: MN-L91+CPT-11<br>Same as group 9 but with i.p. irinotecan<br><br>All irinotecan receiving groups receveived 75mg/kg	1. Histological analysis and mucin staining with PAS/Alcian blue of the jejunum and immunohistochemistry for proliferating cell nuclear antigen (PCNA)<br>2. Intestinal permeability<br>3. Determination of sIgA levels, neutrophil and eosinophil infiltration based on EPO- and MPO-based activity, and cytokine levels in the jejunum<br>4. Determination of SN-38 in intestinal fluid	GF vs CV<br>1. Group 4 intestinal damage ↑ compared to group 5. Proliferation rate ↑ of the intestinal epithelium and ↓ in goblet cells per area of intestinal mucosa in group 4 compared to group 1.<br>2. Group 4 ↑ intestinal permeability compared to groups 1 and 2.<br>3. Neutrophil and eosinophil infiltration ↑ in group 4 compared to group 1. No alterations between groups 2 and 5. Production of pro-inflammatory cytokines (IL-1β and TNF-α) ↑ in group 4.<br>4. SN-38 concentration ↑ in group 5 compared to group 4.<br>CVO vs CV and GF<br>1. Proliferation rate ↑ of the intestinal epithelium and ↓ in goblet cells per area of intestinal mucosa in group 6 compared to group 3.<br>2. Group 6 ↑ intestinal permeability compared to groups 1, 2, and 3.<br>3. Neutrophil and eosinophil infiltration ↑ in group 6 compared to group 3.<br>4. SN-38 concentration ↓ in group 6 compared to group 5.<br><br>MN-TG1 vs MN-L91<br>1. No differences found in histopathology<br>2. Group 8 ↑ intestinal permeability compared to groups 7, 9 and 10<br>3. No differences were found in neutrophil and eosinophil infiltration.<br><br>Conclusion:<br>- Absence of microbiota in GF mice is associated with ↓ of intestinal inflammation, pathology, and gut permeability after irinotecan treatment.<br>- Intestinal damage observed in CV-CPT-11 mice was due to the presence of gut microbiota.<br>- GUS plays a key role in ↑ intestinal permeability.

Wallace BD et al.(9)	2015	USA	Aims to understand and mitigate the GI toxicity caused by cancer drugs, specifically focusing on the role of microbiome GUS.<br><br>*Sub aim:*<br>Determine how inhibition of microbial GUS activity would affect irinotecan pharmacokinetics and GI damage in mice<br><br>A. Examine the ability of Inhibitor R1 to alleviate irinotecan (CPT-11)-induced diarrhea in mice<br>B. Examine the potential impact of Inhibitor 1 on the plasma levels of CPT-11 and its key metabolites SN-38 and SN-38G.	Controlled experimental study using animal subjects	A.<br>27 Balb/cJ wild-type mice in 3 groups (n=9 per group)<br>Group 1: CPT-11<br>CPT-11 injected (50 mg/kg) i.p. 1x/day in the morning<br>Group 2: CPT-11 + Inhibitor R1<br>CPT-11 was injected (50 mg/kg) i.p. 1x/day in the morning and Inhibitor R1 was gavaged (10 µg/d) 2x/day (every 10 hours).<br>Group 3: CPT-11 + Inhibitor 1<br>CPT-11 injected (50 mg/kg) i.p. 1x/day in the morning and Inhibitor 1 gavaged (10 µg/d) 2x/day (every 10 hours).<br><br>B.<br>Balb/cJ wild-type mice in 2 groups:<br>Group 1: CPT-11<br>CPT-11 50mg/kg i.p.<br>Group 2: CPT-11 + Inhibitor 1<br>CPT-11 50mg/kg i.p. and Inhibitor 1 oral gavage (10 µg 2x/day, 3 days before CPT-11 administration and once at CPT-11 administration)	A.<br>Daily monitoring of body weight, stool consistency, occult blood in stool, and GI symptoms (appetite, bowel movements, mobility, etc.)<br><br>B.<br>Plasma levels of CPT-11 and its key metabolites SN-38 and SN-38G were measured.	A.<br>Group 1: bloody diarrhea 25% on day 8, 60% on day 9, and 100% on day 10<br>Group 2: reduced bloody diarrhea compared to group 1, 20% on day 8, and 40% on day 9, 60% on day 10.<br>Group 3: reduced bloody diarrhea compared to group 1, 20% day 9, and 40% day 10.<br><br>B.<br>The presence of Inhibitor 1 did not significantly alter the levels of CPT-11, SN-38, and SN-38G in the mice.

Ervin SM et al.(10)	2019	USA	1. Unravel the molecular basis of regorafenib-glucuronide processing by human intestinal GUS enzymes and examine the potential inhibition of these enzymes.<br><br>2. Examine the reactivation of regorafenib-glucuronide by gut microbial GUS enzymes present in mammalian intestinal contents	Experimental study using animal subjects	A. 10 healthy male and female C57BL/6 and BALB/c mice, 8-16 weeks old, and human feces from 2 donors.<br><br>B. 3 SPF C57BL/6J mice<br><br>C. 5 GF C57BL/6J mice	A. Inhibition of regorafenib-glucuronide reactivation in fecal samples from mice and human donors with fluorescence assay and HPLC methods up to 4h.<br><br>B. Luminal samples were dissected into the cecum, small intestine, and large intestine, homogenized, and repeated A/B procedure up to 48h.<br><br>Testing three inhibitors with the cecal homogenates of SPF mice (UNC7084, UNC7087, and UNC7159; 20µM) and a negative control compound (UNC7088; up to 100µM)<br><br>C. Dissection of cecum into two parts (cecum + all of its contents and only cecum contents), homogenized and repeated A/B procedure up to 48h.	A. No conversion of regorafenib-glucuronide to regorafenib over 4h in fecal samples.<br><br>B. Activity was seen in every portion GI tract, with little conversion in the small and large intestines after 48h. In cecal extracts almost all regorafenib-glucuronide is converted to regorafenib.<br><br>UNC7088 has no inhibition in cecal mixtures. UNC7084, UNC7087, and UNC7159 inhibited conversion in 2 of the 3 samples. UNC7084 failed to inhibit the conversion of cecal contents of 1 mouse<br><br>C. Neither cecum nor its contents were able to catalyze the reactivation of regorafenib-glucuronide to regorafenib after 48h in GF mice.

Cheng KW et al.(11)	2019	Taiwan	1. Determine the effects of a potent *E. coli* GUS-specific inhibitor TCH-3562 for the potential use in preventing CPT11-induced diarrhea<br><br>2. Assess if TCH-3562's inhibition of intestinal bacterial GUS activity impacts plasma SN-38 levels, potentially affecting CPT-11's anti-tumor effectiveness.<br><br>3. Examine if TCH-3562's blocking of gut bacterial GUS affects CPT-11's effectiveness and its intestinal side effects.	Controlled experimental study using animal subjects	Healthy male Balb/c 6- to 8-week-old mice injected s.c. with murine colon carcinoma CT26 cells (1 × 10^6 cell in 50 µL PBS), housed in controlled conditions and divided into 4 groups:<br>Group 1: Control<br>Received ddWater (~200 µL p.o.) and saline (~100 µL i.p.))<br>Group 2: TCH-3562<br>Received TCH-3562 (3 mg/kg/day p.o.) and saline i.p.<br>Group 3: CPT-11<br>Received ddWater p.o and CPT-11 (50 mg/kg/day i.p)<br>Group 4: TCH-3562 + CPT-11<br>TCH-3562 (3 mg/kg/day p.o.) and CPT-11 (50 mg/kg/day i.p.)<br><br>Day 0: cell injection<br>Day 8: starting TCH-3562<br>Day 10-14: CPT-11	1. Daily monitoring of tumor volume (length × width × height × 0.52), diarrhea severity (graded 0-3), and body weight.<br><br>2. Plasma concentrations of SN38 and SN38G levels were measured by LC/MS/MS (groups 3 and 4 only)	1. Tumor growth was inhibited with a 50% ↓ on day 20 in group 3. Group 4 similar trend.<br>Groups 1 and 2 had no diarrhea. Group 3 diarrhea peaked at day 16-day 18, and ↓ from day 19. Group 4 had diarrhea peak on day 17 and recovered from day 18.<br>Body weight ↓ in group 3 days 13-17 compared to group 1. Co-treatment with TCH-3562 had no significant effect.<br><br>2. SN-38 levels ↓ in both groups, with a slight ↑ at 10h in group 4 after TCH-3562 injection. Similar results for the SN-38G plasma levels. AUC and $t_{1/2}$ were ↑ in both SN-38 and SN-38G levels with additional TCH-3562 treatment

Jariwala PB et al.(12)	2020	USA	1. Identify and quantify the bacterial GUS enzymes from human feces<br><br>2. Identify the exact bacterial GUS enzymes responsible for SN-38 reactivation in the gut<br><br>3. Provide a rationale for differential GUS inhibition between human fecal samples by previously designed piperazine-containing GUS inhibitors	Mixed-method approach, combining both in vitro and in vivo study designs	A.<br>Fecal samples from GF wild-type C57/BL6J mice, 8-10 weeks old and colonized by oral gavage and rectal swabbing with viable WT *E. coli* MG1655 or isogenic GUS mutant<br><br>B.<br>Human male (n=2) and female (n=2) fecal samples purchased from a commercial vendor (BioIVT)	A.<br>As a proof-of-concept for labeling GUS enzymes by the cyclophellitol-based ABP's, fecal samples from a controlled mouse model.<br><br>B.<br>1. Identification and quantification of gut bacterial GUS enzymes from human feces with an ABPP-enabled proteomics pipeline<br><br>2. Integration of ABPP-enabled GUS abundance information with *ex vivo* kinetics to pinpoint the specific GUS enzymes responsible for SN-38 reactivation<br><br>3. Inhibition of specific GUS that is involved in SN-38 reactivation with piperazine-containing small molecule inhibitors. GUS activity assay (4MUG), in triplicate for each dose (0.1µM, 1µM, 10µM)	A.<br>Labeling of bacterial GUS enzymes is possible in a controlled fecal matrix.<br><br>B.<br>1. The developed ABPP-enabled proteomics pipeline was able to identify and quantify functionally active GUS enzymes present in human fecal material.<br><br>2. Gut bacterial Loop 1 GUS enzymes are key mediators of SN-38 reactivation<br>- Strong correlation between the Loop 1 (L1) GUS abundance and rate of SN-38-G hydrolysis when compared to total bacterial GUS abundance<br>- Bacterial L1 GUS enzymes process SN-38-G more efficiently than other GUS enzyme classes<br>- *Eubacterium eligens* GUS (EeGUS) processed SN-38G faster than other tested GUS enzymes.<br><br>3. Piperazine-containing small molecule inhibitors, specifically UNC4917 and UNC10201652, target gut bacterial Loop 1 GUS enzymes<br>- SN-38-G processing was differentially inhibited in all four human fecal extracts using the GUS inhibitors<br>- Strong correlation between the inhibition of SN-38-G processing and the abundance of L1 GUS enzymes

Bhatt AP et al.(13)	2020	USA	1. Investigate irinotecan toxicity in the murine GIT and evaluate the protective effects of a single dose of GUSi on gut epithelial damage.<br><br>2. Assess the degree to which GUS inhibition enhances irinotecan's antitumor effectiveness<br><br>3. Determine how irinotecan and GUSi impact murine gut microbiota composition.	Controlled experimental study using animal subjects	A. Healthy female immune-deficient athymic nude mice, 10 weeks old with a minimum weight of 20g, injected s.c. with Sum149 (BRCA1-mutant) cell line, housed in controlled conditions and randomized into 4 groups (n= not specified):<br>1. control<br>2. GUSi (UNC10201652)<br>1 mg/kg , oral gavage<br>3. irinotecan<br>50 mg/kg i.p. daily<br>4. irinotecan + GUSi<br><br>B. Female FVB-TG(C3-1-Tag)cJeg/JegJ mice, ~16 weeks old, housed in controlled conditions and randomized into 4 groups (see A)<br><br>C. Female FVB/NJ mice, 7-8 weeks old, housed in controlled conditions and randomized into 4 groups (n=3-5 per group) (see A)<br><br>D. Female GF wild-type C57/BL6J mice, 8-10 weeks old, housed in controlled conditions, colonized with viable WT *E. coli* MG1655 or isogenic ΔGUS mutant. Four weeks after colonization injected i.p. 50 mg/kg irinotecan.	A. Xenograft model<br>Monitoring of tumor volume (length x Width^2)/2)), diarrhea severity, and body weight. Histological analysis after euthanasia.<br><br>B. C3-Tag transgenic mice<br>Monitoring of tumor volume (length x Width^2)/2)), diarrhea severity, and body weight. Screening via qPCR.<br><br>C. FVB mice<br>Plasma and luminal contents of small intestine, cecum, and colon were collected at 24h and 120h.<br><br>D. Monoassociation studies<br>Monitoring of weight before and 24h after irinotecan. Fecal samples were collected for 16S rRNA amplicon sequencing. Measurement of intestinal inflammation (lipocalin-2 ELISA) and *in fimo* GUS activity (4MUG)	1.<br>- GUSi alleviates irinotecan-induced diarrhea and weight loss in immune-deficient athymic mice.<br>- Irinotecan ↑ inflammation and crypt damage in the colon when compared with irinotecan + GUSi.<br>- GUSi affords strong protection against irinotecan-induced gut epithelial cell damage in immune-deficient mice.<br>- WT *E. coli* mice ↑ gut damage and inflammation with ↓ intestinal epithelial cell proliferation compared to isogenic ΔGUS mutant mice.<br>- GUSi blocks the ↑ of GUS activity and the ↓ of intestinal epithelial cell proliferation after irinotecan treatment in FVB mice.<br><br>2.<br>- GUSi significantly protects against weight loss and diarrhea in C3Tag transgenic mice.<br>- GUSi ↑ irinotecan-mediated tumor regression, ↑ animal survival, and ↓ GI damage in the immunocompetent C3Tag genetically engineered mouse model.<br><br>3.<br>- In immune-deficient mice, GUSi ↑ gut microbial diversity, significantly ↓ irinotecan-induced Proteobacteria growth, and ↓ Proteobacteria levels in the gut without irinotecan treatment.<br>- Irinotecan-treated growth of Proteobacteria due to an ↑ in the family Enterobacteriaceae, which encodes a GUS operon containing the GUS gene and a glucuronide transporter.<br>- Irinotecan does not induce significant changes in the diversity of the gut microbiota in C3Tag mice with an intact immune system.

DA: Dark Agouti; GUS: β-glucuronidase; GI: gastro-intestinal; GIT: gastro-intestinal tract; H: hours; G: grams; ↓: decrease/lower; ↑: increase/higher; PAS: Periodic Acid Schiffs; AT: all timepoints; i.p.: intraperitoneally; H&E: hematoxylin and eosin; w/v: weight/volume; GF: germ free; CV: conventional; EPO: eosinophil peroxidase; MPO: myeloperoxidase; SPF: specific pathogen free; ABPP: Activity-based protein profiling; GUSi: GUS inhibitor

References

1. Kim DH, Jin YH. Intestinal bacterial beta-glucuronidase activity of patients with colon cancer. Arch Pharm Res **2001**;24:564-7
2. Li Y, Zhang X, Wang L, Zhou Y, Hassan JS, Li M. Distribution and gene mutation of enteric flora carrying β-glucuronidase among patients with colorectal cancer. Int J Clin Exp Med **2015**;8:5310-6
3. Zhang J, Lacroix C, Wortmann E, Ruscheweyh HJ, Sunagawa S, Sturla SJ*, et al.* Gut microbial beta-glucuronidase and glycerol/diol dehydratase activity contribute to dietary heterocyclic amine biotransformation. BMC Microbiol **2019**;19:99
4. Stringer AM, Gibson RJ, Logan RM, Bowen JM, Yeoh AS, Keefe DM. Faecal microflora and beta-glucuronidase expression are altered in an irinotecan-induced diarrhea model in rats. Cancer Biol Ther **2008**;7:1919-25
5. Stringer AM, Gibson RJ, Bowen JM, Logan RM, Ashton K, Yeoh AS*, et al.* Irinotecan-induced mucositis manifesting as diarrhoea corresponds with an amended intestinal flora and mucin profile. Int J Exp Pathol **2009**;90:489-99
6. Wallace BD, Wang H, Lane KT, Scott JE, Orans J, Koo JS*, et al.* Alleviating cancer drug toxicity by inhibiting a bacterial enzyme. Science **2010**;330:831-5
7. Roberts AB, Wallace BD, Venkatesh MK, Mani S, Redinbo MR. Molecular insights into microbial β-glucuronidase inhibition to abrogate CPT-11 toxicity. Mol Pharmacol **2013**;84:208-17
8. Pedroso S, Vieira AT, Bastos RW, Oliveira JS, Cartelle CT, Arantes RME*, et al.* Evaluation of mucositis induced by irinotecan after microbial colonization in germ-free mice. Microbiology (Reading) **2015**;161:1950-60
9. Wallace BD, Roberts AB, Pollet RM, Ingle JD, Biernat KA, Pellock SJ*, et al.* Structure and Inhibition of Microbiome beta-Glucuronidases Essential to the Alleviation of Cancer Drug Toxicity. Chem Biol **2015**;22:1238-49
10. Ervin SM, Li H, Lim L, Roberts LR, Liang X, Mani S*, et al.* Gut microbial beta-glucuronidases reactivate estrogens as components of the estrobolome that reactivate estrogens. J Biol Chem **2019**;294:18586-99
11. Cheng KW, Tseng CH, Tzeng CC, Leu YL, Cheng TC, Wang JY*, et al.* Pharmacological inhibition of bacterial beta-glucuronidase prevents irinotecan-induced diarrhea without impairing its antitumor efficacy in vivo. Pharmacological research **2019**;139:41-9
12. Jariwala PB, Pellock SJ, Goldfarb D, Cloer EW, Artola M, Simpson JB*, et al.* Discovering the Microbial Enzymes Driving Drug Toxicity with Activity-Based Protein Profiling. ACS Chem Biol **2020**;15:217-25
13. Bhatt AP, Pellock SJ, Biernat KA, Walton WG, Wallace BD, Creekmore BC*, et al.* Targeted inhibition of gut bacterial beta-glucuronidase activity enhances anticancer drug efficacy. Proc Natl Acad Sci U S A **2020**;117:7374-81
